# A Mobile Game (Safe City) Designed to Promote Children’s Safety Knowledge and Behaviors: Protocol for a Randomized Controlled Trial

**DOI:** 10.2196/17756

**Published:** 2020-06-12

**Authors:** Rosa S Wong, Keith TS Tung, Hiu Tung Wong, Frederick KW Ho, Hing Sang Wong, King-Wa Fu, Ting Chuen Pong, Ko Ling Chan, Chun Bong Chow, Patrick Ip

**Affiliations:** 1 Department of Paediatrics & Adolescent Medicine The University of Hong Kong Hong Kong China (Hong Kong); 2 Institute of Health and Wellbeing University of Glasgow Glasgow United Kingdom; 3 Journalism and Media Studies Centre University of Hong Kong Hong Kong China (Hong Kong); 4 Department of Computer Science & Engineering The Hong Kong University of Science and Technology Hong Kong China (Hong Kong); 5 Department of Applied Social Sciences The Hong Kong Polytechnic University Hong Kong China (Hong Kong)

**Keywords:** serious game, safety training, mobile game, mobile phone, injury prevention, randomized controlled trial, game-based intervention

## Abstract

**Background:**

Children have high levels of curiosity and eagerness to explore. This makes them more vulnerable to danger and hazards, and they thus have a higher risk of injury. Safety education such as teaching safety rules and tips is vital to prevent children from injuries. Although game-based approaches have the potential to capture children’s attention and sustain their interest in learning, whether these new instructional approaches are more effective than traditional approaches in delivering safety messages to children remains uncertain.

**Objective:**

The aim of this study is to test the effectiveness of a game-based intervention in promoting safety knowledge and behaviors among Hong Kong school children in Grades 4-6. It will also examine the potential effect of the game-based intervention on these children’s functioning and psychosocial difficulties.

**Methods:**

This study comprises the development of a city-based role-playing game Safe City, where players are immersed as safety inspectors to prevent dangerous situations and promote safety behavior in a virtual city environment. The usability and acceptability tests will be conducted with children in Grades 4-6 who will trial the gameplay on a mobile phone. Adjustments will be made based on their feedback. A 4-week randomized controlled trial with children studying in Grades 4-6 in Hong Kong elementary schools will be conducted to assess the effectiveness of the Safe City game–based intervention. In this trial, 504 children will play Safe City, and 504 children will receive traditional instructional materials (electronic and printed safety information). The evaluation will be conducted using both child self-report and parent proxy-report data. Specifically, child safety knowledge and behaviors will be assessed by a questionnaire involving items on knowledge and behaviors, respectively, for home safety, road safety, and sport-related safety; child functioning will be assessed by PedsQL Generic Core Scales; and psychosocial difficulties will be assessed by the Strength and Difficulties Questionnaire. These questionnaires will be administered at 3 time points: before, 1 month, and 3 months after the intervention. Game usage statistics will also be reviewed.

**Results:**

This project was funded in September 2019. The design and development of the Safe City game are currently under way. Recruitment and data collection will begin from September 2020 and will continue up to March 1, 2021. Full analysis will be conducted after the end of the data collection period.

**Conclusions:**

If the Safe City game is found to be an effective tool to deliver safety education, it could be used to promote safety in children in the community and upgraded to incorporate more health-related topics to support education and empowerment for the larger public.

**Trial Registration:**

ClinicalTrials.gov NCT04096196; https://clinicaltrials.gov/ct2/show/NCT04096196

**International Registered Report Identifier (IRRID):**

PRR1-10.2196/17756

## Introduction

### Background

Injury is the leading cause of death and disability among children in many parts of the world [1,2]. Despite the decreasing trend of injuries around the world, the burden of injury remains high in Hong Kong. Cross-cultural research showed that the rate of unintentional transport-related injury was higher in Hong Kong than in other developed countries [3,4]. Between 2001 and 2012, 742,552 children and adolescents in Hong Kong were treated for injuries including unintentional injuries attributable to home accidents (39%), sports (18%), and road traffic (4%), resulting in an annual direct medical cost of HK $230 million (US $29.4 million) and an indirect cost of up to 2-3 times higher [5]. Although traffic injuries were more common in younger children, domestic and sport injuries were among the top 3 leading causes of injury in the age groups of 5-9 years, 10-14 years, and 15-19 years, respectively [5]. Furthermore, during the same study period, more than 753 children died from injuries, with traffic injury as the most frequent cause (0.69 deaths per 100,000 people) [5]. These findings suggest that injuries can substantially cost society and should be prevented as early as possible.

Despite the potentially severe and unplanned nature of unintentional injury, these problems are largely predictable and preventable. In Hong Kong, some preventive efforts have been made by the government and health care professionals. For example, the Road Safety Town and Bus were developed to provide a simulated road environment to increase local children’s awareness of road safety [6]. Online resources and smartphone apps for prevention of children’s sport and domestic injuries are also available in Hong Kong [7]. Nonetheless, these programs and resources are often time bound and designed to guide mainly parents or teachers to minimize the injury hazards for children. Local Hong Kong children lack opportunities to learn safety and apply the safety knowledge by themselves in real-life situations. There are also limited resources for children to learn safety outside of school. Traditional classroom-based education often uses one-way instructional approach from the instructor to the students, which can make learning dull and ineffective [8].

In recent years, game-based learning has become increasingly popular. Some evidence shows that game-based learning can enhance student motivation and engagement and critical thinking skills [9]. It can also trigger curiosity and fantasy in students, thereby increasing their intrinsic motivation and enjoyment in learning [10]. For example, serious games, defined as computerized games produced for educational or training purpose [11], can provide a platform for children to learn and explore the materials at their own pace. After gameplay, teachers can conduct in-class discussions on students’ gaming experiences to reinforce the new knowledge gained from the gameplay. Teachers can also make use of the in-game point and ranking system to develop healthy competition between students to make learning more interesting. Several meta-analyses have shown support for the positive effects of video games with elements such as immersive stories and interactive game environment on health-related behavior including diet, physical activity, and symptom management [12,13]. By contrast, there has been inconsistent evidence on the association between time spent playing games and psychosocial difficulties such as social, emotional, and behavioral problems in children [14,15]. Several studies have shown a relationship between violent video game use and aggressive behavior and poor performance in school [14,16]. However, little is known about the impact of serious games on children’s functioning and psychosocial difficulties.

Based on the experiential gaming model proposed by Kiili [17], positive user experiences are reinforced through the use of immediate feedback, clear goals, and developmentally appropriate challenges. Experiential learning is also known as the process of learning through experience, which has demonstrated effectiveness in enhancing student reasoning and critical thinking skills [18]. Most of the existing safety training studies were conducted through virtual reality (VR) games, which allow children to learn and practice safety techniques in realistic simulated situations. A meta-analysis published in 2014 identified 19 articles on child pedestrian safety behavioral interventions and found that repeated practice in vivo at street-side locations or in game-based VR environments was the most effective strategy [19]. Although VR techniques are increasingly used for education and training purposes, game-based VR training requires expensive production, and young children without adequate safety knowledge may find it difficult to navigate the virtual environments alone [20]. To overcome these VR game limitations, another popular genre of video and computer games introduced is role-playing games (RPGs). As with VR game, RPG allows players to strategize and interact with in-game objects and resources, thereby increasing their motivation, critical thinking, and problem-solving skills [21,22]. Instead of training players in realistic simulated hazard situations, RPG players can create their own avatar/character customized with unique attributes, skills, and traits to play and advance in the cyber world. However, no trials have been conducted to evaluate the effectiveness of RPG-based intervention for injury prevention.

To our knowledge, only few digital safety games are available on the market, all of which have high usage and download rates but are developed for Western child populations and in English language [9,23]. Although there has been positive user feedback on these games, including better safety knowledge and behaviors [23], cultural and language differences could undermine non–English-speaking children’s interest in these games. Hence, we would like to design and develop a Chinese digital safety game for Hong Kong children.

### Aims of This Study

This paper describes the protocol for a study that will test the effectiveness of an intervention using a mobile city game, Safe City, to improve the safety knowledge and behaviors of Hong Kong Chinese children in Grades 4-6 by a randomized controlled trial. Our hypotheses are as follows: (1) children in the intervention group (ie, those who receive the Safe City game intervention) will have higher levels of correct safety knowledge and behaviors than children in the control group (ie, those who receive electronic and printed safety information) at 1- and 3-month follow-ups. (2) We expect a change in the children’s functioning and psychosocial difficulties. At 1- and 3-month follow-ups, we expect that children in the Safe City intervention group will show better functions and fewer psychosocial difficulties than those in the control group. (3) We expect that those who play the game more frequently will show greater improvement in safety- and health-related outcomes.

## Methods

### Research Strategy

This study will follow the analysis, design, development, implementation, and evaluation model of instructional design [24] to design, produce, and test a mobile safety game–based intervention for improving safety knowledge and behaviors in Hong Kong Chinese children.

### Game Design

The mobile game, Safe City, will be co-designed by our project team (pediatricians, psychologists, safety experts, and experienced health promotion researchers) and the game development team (programmers, artists, and sound designers). The model of learning used in the Safe City game will be guided by the social cognitive theory [25] and elaboration likelihood model [26]. The elaboration likelihood model proposes that capturing attention is the first step in motivating people to process information [26], whereas the social cognitive theory proposes that behavioral change is a function of improved skills and confidence through repeated modeling, feedback, or both [25]. Hence, the Safe City game will be packaged as an RPG with multiple components to teach players how to recognize and minimize injury hazards to prevent injuries in a real-world environment.

In the game, players will start by indicating their preferred gender, hairstyle, facial features, and clothing for their human avatar who will assume the role of safety inspector in the virtual city. This avatar-creation approach is to engage the player in the game as if he or she is exploring the game city using the first-person perspective. As a safety inspector, the player will use the city map provided in the game to identify safe routes to navigate between potentially dangerous checkpoints (homes and athletic areas) and assess the safety level of the checkpoints in the busy and lively land of Safe City. The gameplay will feature mini games (eg, multiple choice questions, spot the danger, and matching items to reinforce safety messages) with fun elements such as game points and in-game ranking to encourage daily play of the game plus engagement strategies such as positive reinforcement (ie, point addition for each correct response) and response cost (ie, point deduction for each incorrect response) to promote safety behavior.

**Figure 1:**
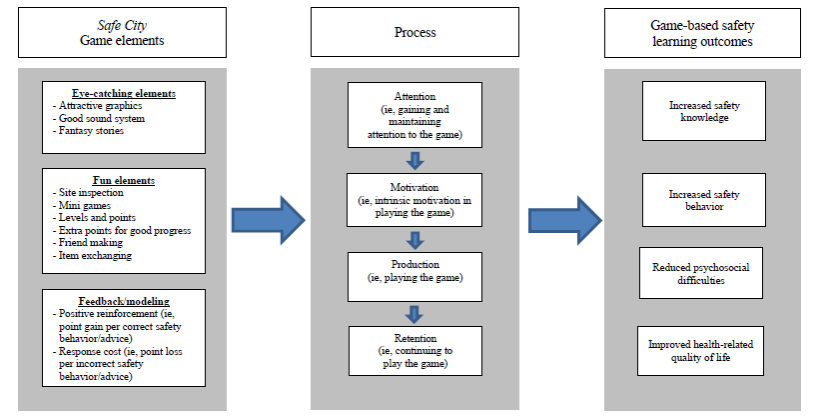
The Safe City gaming process.

**Figure 2:**
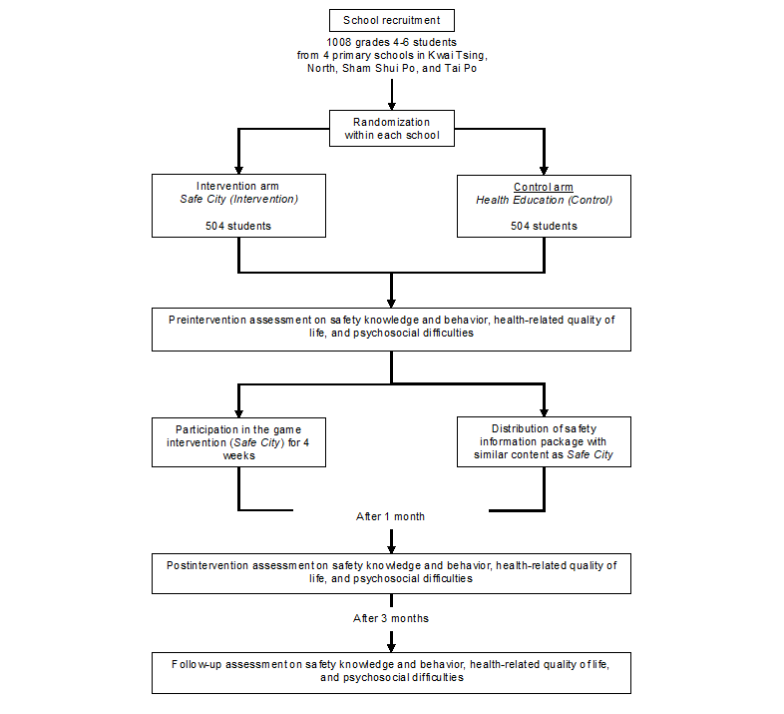
Study flowchart.

Figure 1 illustrates the gaming process by which the Safe City game–based intervention may influence the students’ target outcomes. All these game concepts and technical specifications of the game will be stated in the game design document for guiding the development of prototypes of the game. To create an effective and developmentally appropriate learning product, a multiple-phase study approach (collaboration, prototyping, and evaluation) will be employed to produce design prototypes that are developmentally appropriate, comprehensible, and attractive for the end users (children in Grades 4-6) [27]. Collaboration will involve working with game designers and exploring ideas for design prototypes. The prototype game will be played by a group of 4-8 children from the target age group and a debriefing session will be arranged to review their gameplay experiences. The gameplay will be further adjusted based on these end users’ feedback. During the evaluation phase, a 4-week, 2-arm randomized intervention trial will be conducted with 3 data collection periods (baseline and 1- and 3-month follow-ups). Participants will be randomized into 2 groups (the Safe City game intervention group and a traditional health education group) in a 1:1 allocation ratio (Figure 2).

### Participants

Four districts, including Kwai Tsing, North, Sham Shui Po, and Tai Po, were found to have the highest rates of traffic, domestic, and sport injuries in Hong Kong [5]. All nonspecial, local (noninternational) primary schools in the selected districts will be eligible for participation in the evaluation. Among them, we will recruit 4 schools. Assuming an average of 30 students per class and a participation rate of 70%, the trial will have 4 schools × 3 forms × 4 classes × 30 students × 70% = 1008 participants. Primary schools will be a blocking factor in randomization. All students in Grades 4-6 will be invited to join the trial. Participating students within each school will be randomized with a 1:1 allocation ratio into the intervention or control group. Because the intervention materials will be designed in traditional Chinese, students who are not able to comprehend the Chinese questionnaires at baseline will be excluded from this study. An invitation letter will be sent to all schools in the selected districts. Upon obtaining school consents, informed written consents will be collected from parents of participating students before randomization.

### Sample Size Calculation

A previous intervention study reported a small-to-medium effect on road crossing behaviors (Cohen *d*=0.258) [23]. Assuming a conservative effect of 0.181 (70% of the previous study), 1008 participants (504 in each group) should be recruited to detect this effect size with 80% statistical power at 5% significance level.

### Intervention

A total of 1008 Hong Kong Chinese children studying in Grades 4-6 will be recruited and randomly allocated to the intervention (ie, the Safe City game) and control (ie, promotional leaflets on safety information) groups. The participant recruitment and randomization process will be independently carried out by different research assistants. Outcome assessors will be blinded to the allocation of participants in each group. To avoid contamination between groups, unique login credentials will be provided to each participant. The participant will need to enter these login credentials when accessing the game. This strategy is designed to prevent students in the control group from getting the same intervention. At follow-ups, a questionnaire item will ask whether the participant has used the Safe City game to inform subsequent analyses.

Students in the intervention group will be given a manual containing instructions to play the Safe City game. A research assistant will provide a briefing session on the game in each participating school. A unique username and password set will be created for each user to log in the game. These login credentials will be provided to the student participants in a sealed envelope after the briefing session and can be retrieved from the research team anytime during the intervention period. The participants will be permitted to play the game as many times as desired within a 4-week timeframe. The players ranked in the top 20 will receive a reward in the form of book coupon after the intervention ends.

All students in the control (ie, health education) group will receive a comprehensive package on safety information. The information package includes both printed and electronic promotional materials regarding safety and a comprehensive list of relevant website and information sources. The information from these relevant websites and information sources is similar to that used in developing the safety case scenarios for the Safe City game. Consequently, both intervention and control arms will have comparable accessibility to safety-related information, and the major contrast between the two groups will be the method of presentation (game-based learning vs traditional health promotion approach, ie, unidirectional information package).

### Outcomes

#### Primary Outcomes: Safety Knowledge and Behaviors

Child safety knowledge and behaviors will be measured by a questionnaire involving items adopted from the existing literature and questionnaires under the 3 contexts: home safety [28], road safety [23], and sport-related safety [29]. Some item wordings will be modified to ensure their relevance to local environments. Child participants will be asked to indicate their level of involvement in the said behavior (eg, home safety: *use sharps without the presence of my parents*; road safety: *forget to look properly because you are thinking about someone else*; sport-related safety: *play while injured*) on a 5-point Likert scale ranging from “Never” to “Very Often.” They will also be asked to indicate their agreement on safety-related statements (eg, home safety: *never leave food or water unattended on the stove*; road safety: *stop at red lights and stop signs*; sport-related safety: *do warm-up exercise before having any sports activities*) on a 4-point Likert scale ranging from 1=Strongly disagree to 4=Strongly agree. A total score will be generated for each safety context by summing the relevant items. These items will be self-completed by the children at baseline and 1- and 3-month follow-ups. At each follow-up, their parents will also be asked to rate the degree to which children have shown changes in safety knowledge and behavior after the intervention on a 5-point Likert scale ranging from 1=Much worse to 5=Much better.

#### Secondary Outcomes

##### PedsQL Generic Core Scales

Child functioning will be measured by both child self-report and parent proxy-report versions of the Chinese PedsQL Generic Core Scales which are suitable for use in ages 8-12 [30]. It has 4 scales with 23 items measuring child physical (eg, I hurt or ache), emotional (eg, I feel afraid or scared), social (eg, I have trouble getting along with other kids), and school functioning (eg, I miss school because of not feeling well). Each item is rated on a 5-point scale from 0 (never) to 4 (almost always). A higher score indicates better functioning. Its Chinese version has been validated in Hong Kong with good psychometric properties [31].

##### Strength and Difficulties Questionnaire

Both child self-report and parent proxy-report versions of the Chinese Strength and Difficulties Questionnaire (SDQ) will be used to measure children’s psychosocial behavior. The SDQ has 5 subscales, including emotional symptoms (5 items; eg, often unhappy, depressed, or tearful), conduct problems (5 items; eg, often loses temper), hyperactivity/inattention (5 items; eg, restless, overactive, cannot stay still for long), peer relationship problems (5 items; eg, rather solitary, prefers to play alone), and prosocial behavior (5 items; eg, considerate of other people’s feelings). Each subscale has a score, and the first 4 subscales generate a total score of difficulties (20 items) [32]. The parent proxy-report version of SDQ in traditional Chinese has been validated and demonstrated satisfactory reliability and validity [33]. The self-reported version has also been used in younger children (aged 8-13 years) with satisfactory results [34].

##### Game Usage Statistics

The user account system will capture all in-app/task-specific actions taken by the player, including login time, gameplay duration, total game points, the number of correct and incorrect responses in each safety domain (ie, home safety, road safety, and sport-related safety), and the number of attempts needed to reach the correct answer. The account system will only be accessible to the members in the research team, and all user data will be encrypted with a unique password generated by the research team. The usage data will be retrieved from the account system for process evaluation as well as for guiding award reimbursement.

### Data Analysis

Results will be reported from intention-to-treat analysis, where outcome variables of the dropouts will be assumed to be unchanged from the previous assessments to provide a conservative estimate. A complete-case scenario will also be considered as a sensitivity analysis. The effectiveness of the intervention in achieving the proposed targets will be estimated using linear mixed models. Group allocation will be included as dummy independent variable. School will be controlled as random intercepts, whereas covariates, such as child’s age and sex and family socioeconomic status, will be adjusted as fixed independent variables. The linear model will be used for continuous outcomes, whereas logistic and Poisson models will be used for binary and count variables, respectively. The association between total game points and pre–post changes in outcomes will also be analyzed using the linear mixed models among students in the intervention group. Students in the control group will not be considered in this analysis because they will not be able to access the game. Game usage statistics will be added to the model as independent variables.

### Ethical Issues

This trial is registered on the ClinicalTrials.gov database (NCT04096196) and has been approved by the Institutional Review Board of Hong Kong University and Hospital Authority Hong Kong West Cluster (Reference number: UW 19-028).

## Results

Safe City is currently at the initial stages of development and programming of its prototype version. Acceptability and usability tests on target participants will subsequently be conducted to detect and correct occasional bugs or problems and refine the design. Participant recruitment will commence in September 2020. Completion of data collection is anticipated to occur in March 2021, and analysis of results will be undertaken by December 2021.

## Discussion

Childhood injury is a major public health problem. Although it is the leading cause of disability and death for children in many parts of the world [1,2], prevention strategies to tackle this public health problem are still inadequate. Health education is an important part of health promotion, and middle childhood is the ideal time for health education. In Hong Kong, safety training and programs often take place in formal education settings such as classroom. Although simulated road facilities such as Road Safety Town and Bus are available in the local community for children to learn road safety, this type of community-based learning has limitations such as short visit time and inconvenient location. Recent advances in technology allow students to learn with more opportunities to explore real-world problems and challenges in a virtual environment [9]. Evidence suggests that game-based learning may enhance positive learner experiences, feelings of empowerment and self-efficacy, and positive psychological outcomes including enjoyment and confidence [12,35,36]. Several safety games have been developed for Western children with high usage and download rates [9,23]. This will be the first report of a safety promotion game developed for Hong Kong child population. We are hopeful that the game can be a valuable tool for prevention of childhood injury in the community in the long run. Moreover, the resulting city game platform can be used for general health education purpose, so other health topics such as mental health and substance use can also be incorporated into the game in future development phases. Moreover, this is the first known research trial assessing the effectiveness of a mobile serious game in teaching children about safety knowledge and behaviors. Findings from this study will be made available to the public, local schools, news media, education groups, and safety organizations working to improve injury prevention in Hong Kong and beyond, as they could inform future innovative strategies for injury prevention.

## Appendix

Multimedia Appendix 1 Peer-reviewed reports of the funding agency.

## Abbreviations

RPG: role-playing game
VR: virtual reality
