# Supplementary material for: A Mobile Game (Safe City) Designed to Promote Children’s Safety Knowledge and Behaviors: Protocol for a Randomized Controlled Trial
Source: JMIR Res Protoc. 2020 Jun 12;9(6):e17756. doi: 10.2196/17756 (PMC7320307; doi:10.2196/17756)
Supplement: Multimedia Appendix 1 [file resprot_v9i6e17756_app1.pdf]

The point by point response to the Grant Review Board and Reviewers' comments

Project No: **02180768**
Project Title: **Safe City: a game-based educational approach to promote child safety knowledge and behaviours**
Principal Applicant (PA): **Dr. Patrick IP**

GRB Comments

**GRB(1):**	**The project is well written.**
A:	*Thank you very much for the comment.*

**GRB(2):**	**Elaborate the scientific evidence of the effectiveness of the proposed intervention on the target group.**
A:	*Thank you for the suggestion. We have elaborated more in Section 13(b) (ii) and (iii).*<br><br>*Digital games have been increasingly used for health promotion. A review of 25 articles through December 2006 identified 25 video games which produced positive changes in health-related behavior including diet, physical activity and symptom management through various game elements such as immersive stories and interactive game environment (1). As mentioned in our original proposal, to our knowledge, there are only few digital safety games available on the market, all of which are developed for western children and in English language. Scientific evidence on the effectiveness of these safety games are scant as agreed by Reviewer 1 and Reviewer 2. In view of this research gap and the growing use of digital games for education and training, this proposed project will develop a 2D online city game with role-playing game (RPG) techniques, mini-games and other game elements that foster interactions between players. Its effectiveness as part of the safety education for Hong Kong Chinese young children will be evaluated through a randomized controlled trial (RCT) study design.*<br><br>*Reviewer 3 pointed out that we did not give a thorough literature on training games in our original proposal. This is because most of the existing safety training studies were conducted through virtual reality games which allow children to learn and practice safety techniques in realistic simulated situations. A meta-analysis published in 2014 identified 19 articles on behavioral child pedestrian safety interventions and found that repeated practice in vivo at street side locations or in game-based virtual reality environments were the most effective strategies (2). A RCT study compared the effectiveness of three strategies of pedestrian safety training (street side/field, video/websites and virtual reality (VR) training) in improving pedestrian safety skills among 7- and 8-year-old children (3). The study found that children trained in VR and field environments had safer pedestrian behavior than control group children at post-intervention and at follow-ups.*<br><br>*While VR techniques are increasingly used for education and training purposes*

	because of their advantages on risk, acceptance and effectiveness over previous street side/field training methods, game-based VR training requires expensive production and can be difficult for young children as they may have not yet learned the safe ways to navigate environments alone (4). To overcome these VR game limitations, another popular genre of video and computer games is role-playing games (RPG) which gives the game developer more flexibility in designing developmentally appropriate adventure story with attractive characters and solvable challenges to engage the young participants. Like VR game, RPG allows players to strategize and interacts with objects and resources, but instead of training players in realistic simulated hazard situations, RPG players create their own avatar/character customized with unique attributes, skills and traits to play and advance in the cyber world. Furthermore, RPG can be combined with social game elements such as chat and items exchange to foster collaboration and communication among players (5). Some of these games are referred as massively multiplayer online role-playing game (MMORPG). RPGs or MMORPGs can facilitate learning by increasing student motivation and critical thinking and problem-solving skills (6, 7). Preliminary evidence showed that MMORPGs can lead to greater improvement in English communicative skills than traditional teaching method (8). In a recently published pilot RCT on HIV prevention, young adolescents participating in the smartphone game-based intervention with RPG techniques showed a larger gain in sexual health-related knowledge and self-efficacy compared with the control group (9). However, there is a lack of robust scientific evidence on RPG for injury prevention, highlighting the need to conduct this proposed project which will be able to demonstrate whether RPG can be used as a health promotion tool to prevent injury and potentially other health risk behaviors in Hong Kong.<br><br>*References:*<br>*1. Baranowski, T., Buday, R., Thompson, D. I., & Baranowski, J. (2008). Playing for real: video games and stories for health-related behavior change. American journal of preventive medicine, 34(1), 74-82.*<br>*2. Schwebel, D. C., Barton, B. K., Shen, J., Wells, H. L., Bogar, A., Heath, G., & McCullough, D. (2014). Systematic review and meta-analysis of behavioral interventions to improve child pedestrian safety. Journal of pediatric psychology, 39(8), 826-845.*<br>*3. Schwebel, D. C., McClure, L. A., & Severson, J. (2014). Teaching children to cross streets safely: A randomized, controlled trial. Health Psychology, 33(7), 628.*<br>*4. Smith, S., & Ericson, E. (2009). Using immersive game-based virtual reality to teach fire-safety skills to children. Virtual reality, 13(2), 87-99.*<br>*5. Dickey, M. D. (2007). Game design and learning: A conjectural analysis of how massively multiple online role-playing games (MMORPGs) foster intrinsic motivation. Educational Technology Research and Development, 55(3), 253-273.*<br>*6. Umaschi Bers, M. (2001). Identity construction environments: Developing personal and moral values through the design of a virtual city. The Journal of the Learning Sciences, 10(4), 365-415.*<br>*7. Childress, M. D., & Braswell, R. (2006). Using massively multiplayer online role-playing games for online learning. Distance Education, 27(2), 187-196.*<br>*8. Suh, S., Kim, S. W., & Kim, N. J. (2010). Effectiveness of MMORPG-based instruction in elementary English education in Korea. Journal of computer*

<table>
<tr><td></td><td>assisted learning, 26(5), 370-378.
9. Winskell, K., Sabben, G., Akelo, V., Ondeng'e, K., Obong'o, C., Stephenson, R., ... & Mudhune, V. (2018). A smartphone game-based intervention (Tumaini) to prevent HIV among young Africans: pilot randomized controlled trial. JMIR mHealth and uHealth, 6(8).</td></tr>
</table>

<table>
<tr><td>GRB(3):</td><td>Elaborate the design of the game and provide content of the game such as interface and screenshots.</td></tr>
<tr><td>A:</td><td>Safe City is a 2D online city game that provides a multicomponent approach (e.g. role-playing, mini-games and interactive environment) to enhance learning through design grounded in the principles of safety education pedagogy. Guided by social cognitive theory and elaboration likelihood model, the model of learning used in Safe City incorporates various game elements to enhance player attention (through eye-catching elements), motivation (through fun elements), production and retention (through modelling and/or feedback).

<u>Proposed Safe City model</u>

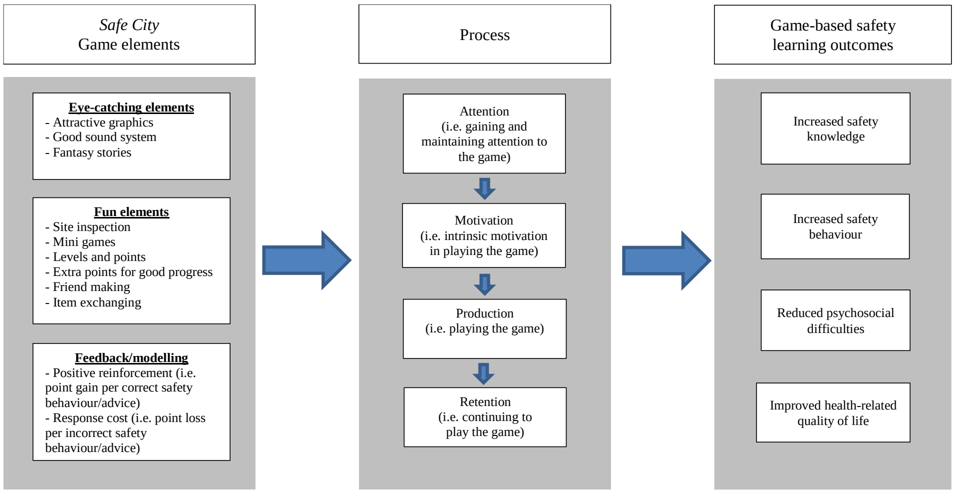

Further details on the game's design and content such as sample screenshots can be found in the Annex 2 of the revised proposal.</td></tr>
</table>

<table>
<tr><td>GRB(4):</td><td>How to avoid the problem of contamination within schools?</td></tr>
<tr><td>A:</td><td>Thank you for pointing out the problem of potential contamination between intervention and control group. Since the main intervention is delivered through a web/app-based platform, we will provide unique login credentials for students randomized into the intervention group. Such unique login credentials will be required to access the game and participants would be advised not to share and exchange such information with their peers. This information has been added in the revised proposal (clean version, P.6). Under this scheme, we are confident that contamination would be minimized. To evaluate the degree of contamination, we will also ask in the questionnaire whether the students in the control group accessed the game through other peers' login credentials. Sensitivity per protocol analysis will be conducted using this information.</td></tr>
</table>

GRB(5):	**Elaborate the sustainability of the proposed project.**
A:	*The Safe City game produced by this project shall be made available to the public and local schools and shall continue to run under the management and maintenance by the Department of Paediatrics and Adolescent Medicine of the University of Hong Kong after the expiry of the funding support from the Health Care and Promotion Scheme.*<br><br>*Upon completion of this project, flyers about the Safe City game will be made and distributed in seminars and workshops as well as sent to all local schools, news media, education groups and safety organizations to publicize the game and increase its usage in the community. Information about the game will also be posted in social networking websites (e.g. Facebook). Safe City evaluation results will be shared in various meetings, conferences and events to raise public awareness and usage of the game. This information has been added in the revised proposal (clean version, P.11).*<br><br>*Meanwhile, we would continue to seek funding for upgrading the game to serve other health education purposes and for use in multiple platforms.*

GRB(6):	**An ethics approval from a recognized ethics committee is required**
A:	*We are in process of applying for the ethics approval. The scanned copy of the ethics approval will be submitted to the GRB by email as specified in the decision letter.*

GRB(7):	**Clarify if the following budget item: "Incentives for participants" ($9,200) should be re-named as "Award for participants".**
A:	*Thank you very much for your suggestion. The following item "Incentives for participants" have been re-named as "Award for participants".*

GRB(8):	**Provide justification and breakdown for the following budget item: "Game design and development" ($780,000).**
A:	*Thank you very much for your comments. Justification and breakdown of the budget item "Game design and development" has been added, as below:*<br>***Game programming: $270,000***<br>*A game programmer will be hired for 12 months to develop codebase for the proposed game.*<br>***Gaming component designing: $280,000***<br>*A game designer will be hired for 12 months to design the game content including creating goals, rules, challenges, game point allocation, level system that could produce a desirable gaming environment for the users to interact and engage in the game.*<br>***Graphics or Art design: $200,000***<br>*A game artist will be hired for 10 months to create 2D art for the visual elements of a video game, such as characters, vehicles, props, scenery, background, objects, colors, textures, and clothing.*<br>***Game sound design: $30,000***<br>*A sound designer will be hired at the final stage of the game design to generate and adjust audio elements for the game.*

Reviewer 1

**R1(1):**	**Promoting children's safety knowledge and behaviors via a game-based approach is novel. The proposed work is highly relevant to the thematic priority "Injury prevention".**
A:	*Thank you very much for your comments.*

**R1(2):**	**The aims and objectives are clearly defined. They are well informed by scientific evidence.**<br><br>**As the applicants mentioned, there are quite limited number of digital safety games on the market. But it is useful to provide more evidence support for using the digital games for enhancing health-related knowledge and behaviors (other than safety).**
A:	*Thank you very much for your comments. We have elaborated the scientific evidence on digital games in section (b) (ii) and (iii).*

**R1(3a):**	**The implementation plan is detailed and appropriate for the project.**<br><br>**Sample size has been adequately justified.**<br><br>**The proposed timeline is feasible.**
A:	*Thanks for your comments.*

**R1(3b):**	**The only concern is the potential problem of contamination. The same school has students in both intervention and control group. How to ensure that contamination won't happen?**
A:	*Thank you for pointing out the problem of potential contamination between intervention and control group. Since the main intervention is delivered through a web/app-based platform, we will provide unique login credentials for students randomized into the intervention group. Such unique login credentials will be required to access the game and participants would be advised not to shared and exchange such information with their peers. This information has been added in the revised proposal (clean version, P.6). Under this scheme, we are confident that contaminations would be minimized. To evaluate the degree of contamination, we will also ask in the questionnaire whether the students in the control group accessed the game through other peers' login credentials. Sensitivity per protocol analysis will be conducted using this information.*

**R1(4):**	**The indicators are clearly defined and outcome evaluation tools are appropriate for the purposes**
A:	*Thanks for your comments*

**R1(5):**	**The team has a strong track record in health promotion for children and youth.**

	**The team consists of cross-sector collaborations among academic institutions and NGO.**
A:	*Thanks for your comments.*

**R1(6):**	**It was mentioned that the preliminary design for the game has already been completed at the time of the application. Need to clarify whether the budget requested is to cover the remaining work.**
A:	*Thanks for your comments. The preliminary design of the game, including the main goals, mode of gaming, and target audience of the game, was developed by the project team during the application process. However, a professional game development team with expertise in different areas of game development including game programming, game design, graphics designs, and audio design is needed and essential for producing an attractive, playable and immersive game.*

**R1(7):**	**The developed digital game could be used after the completion of this project.**<br><br>**It is not clear how local resources are mobilized to continue usage of the developed game in the community.**
A:	*Thank you for the comment. The Safe City game produced by this project shall be made available to the public and local schools and shall continue to run under the management and maintenance by the Department of Paediatrics and Adolescent Medicine of the University of Hong Kong after the expiry of the funding support from the Health Care and Promotion Scheme.*<br><br>*Upon completion of this project, flyers about the Safe City game will be made and distributed in seminars and workshops as well as sent to all local schools, news media, education groups and safety organizations to publicize the game and increase its usage in the community. Information about the game will also be posted in social networking websites (e.g. Facebook). Safe City evaluation results will be shared in various meetings, conferences and events to raise public awareness and usage of the game. This information has been added in the revised proposal (clean version, P.11).*<br><br>*Meanwhile, we would continue to seek funding for upgrading the game to serve other health education purposes and for use in multiple platforms.*

**R1(8a):**	**Strengths: A well written proposal and a strong team**
A:	*Thank you very much for your comments.*

**R1(8b):**	**Weaknesses: clarify how contamination could be avoided.**
A:	*As described above, we will minimize contamination through unique login credentials for each student in the intervention group and will evaluate the effect of continuation in sensitivity per protocol analysis.*

R1(8c):	**Weaknesses: Strengthen the sustainability plan.**
A:	*The sustainability plan has been strengthened by adding "Upon completion of this project, flyers about the Safe City game will be made and distributed in seminars and workshops as well as sent to all local schools, news media, education groups and safety organizations to publicize the game and increase its usage in the community. Information about the game will also be posted in social networking websites (e.g. Facebook). Safe City evaluation results will be shared in various meetings, conferences and events to raise public awareness and usage of the game" in the revised proposal (clean version, P. 11).*

<u>**Reviewer 2**</u>

**R2(1):**	**The project is innovative in using a digital game based experiential learning approach to promote child safety knowledge and behaviors, addressing injury prevention thematic priority needs among children in HK, rather than the traditional safety programs in formal education settings lacking practice and interactive elements to enhance student learning motivation and behavioral changes.**
A:	*Thank you very much for your comments.*

**R2(2):**	**Aim and objectives were well articulated. The proposed strategy was well supported by related scientific evidence.**
A:	*Thank you very much for your comments.*

**R2(3):**	**The target group is over 1,000 P4-6 children. The implementation plan and milestones are well designed including the content of the city game in achieving its stated objectives.**
A:	*Thank you very much for your comments.*

**R2(4):**	**RCT evaluation plan well designed with control group and well selected indicators, evaluating the 5 specific objectives of the project, comparing the effectiveness of the project approach with traditional strategy in changing children's safety knowledge and behaviours, enhancing their health-related quality, and reducing their psychosocial difficulties**
A:	*Thank you very much for your comments.*

**R2(5):**	**Strong and experienced team from multi-disciplinary background.**
A:	*Thanks for your comments*

**R2(6):**	**Mainly on RA and design & development of the game.**<br><br>**Justified and reasonable.**
A:	*Thanks for your comments*

**R2(7):**	**The game platform could be readily extended to new children users and upgraded in content and design to meet the new safety education needs of the children in our city context.**
A:	*Thanks for your comments*

**R2(8):**	**Strengths:**<br>**1. Innovative in approach.**<br>**2. Addressing limitations of traditional approach**<br>**3. Well planned in implementation and evaluation**<br>**4. Strong and experienced team**
A:	*Thanks for your comments*

R2(9a):	**Weaknesses:**<br>1. **The game appears not interactive among children learners, thus reducing the peer learning effects**
A:	*Thank you for your comments. To foster peer learning, we would add in social game features. For example, players can make friends with other players and exchange items and ideas with each other within the game environment, such that players can work together to solve the problems in the cyber city. This additional game feature has been added in the revised proposal (clean version, P.16).*

R2(9a):	**Weaknesses:**<br>2. **How could it be integrated into the traditional classroom learning approach?**
A:	*E-learning is important for high quality education and training. Safe City as an e-learning resource can promote self- and peer-learning, which in turn could increase and improve student-teacher interactions, as traditional classroom learning focuses on teacher-led activities which may not foster students' critical thinking problem solving and decision making skills, whereas Safe City can let children learn and explore the materials at their own pace. After gameplay, teachers can do in-class discussion on students' gameplay and learning experience (e.g. what kind of knowledge students learned from the game and which safety domains students would like to know more etc) that would be useful for teachers' preparation of subsequent teaching materials. Teachers can also use the Safe City game to make healthy competition between students to make learning more interesting. Furthermore, students can access Safe City an unlimited number of times which could consolidate the materials learned in class. This information has been added in the revised proposal (clean version, P.2).*

Reviewer 3

**R3(1a):**	**The investigators argued that traditional health education approaches are usually classroom-based and use one-way method to deliver the information from the instructor to the audience, however, this is too subjective and lack of literature support. School-based and parent oriented approaches are still the most effective way for children in learning. I don't pretty agree with the team and will still believe that the best way to learn safety is to have it be integrated to the school system and in the family. There does not exist any conflicts with parent-child collaborative decision making as cited in (iii)**
A:	*Thank you for your comment. We have further clarified the rationale for this proposed project in our revised proposal (clean version, section (b) (ii) and (iii)). Briefly, we agree with the reviewer that school-based approach is an effective learning way for children, but as documented in literature, both e-learning and traditional classroom training have their own advantages (e.g. traditional training: being familiar to both teachers and students; e-learning: learner-centered and self-paced) and disadvantages (e.g. traditional learning: time and location constraints; e-learning: lack of teacher-student interaction) (1). Integration of the Safe City game as an e-learning resource into traditional health education would have the potential to complement both approaches. For example, the Safe City game can promote self- and peer-learning, which in turn could increase and improve student-teacher interactions, as traditional classroom learning focuses on teacher-led activities which may not foster students' critical thinking problem solving and decision making skills, whereas Safe City can let children learn and explore the materials at their own pace. After gameplay, teachers can do in-class discussion on students' gameplay and learning experience (e.g. what kind of knowledge students learned from the game and which safety domains students would like to know more etc) that would be useful for teachers' preparation of subsequent teaching materials. Teachers can also use the Safe City game to make healthy competition between students to make learning more interesting. Furthermore, students can access Safe City an unlimited number of times which could consolidate the materials learned in class. Hence, using both approaches could further promote positive learning experiences, especially when there is evidence suggesting that game can increase children's enjoyment in learning more than classroom lessons (2).*<br><br>*In addition, we would like to clarify that our intention is not to devalue or replace parent oriented approaches but to make them more effective in helping children to learn by integrating an e-learning resource in the family. As stated in the original proposal, research shows that parent-child collaborative decision making could facilitate parental management of child issues, including, but not limited to, reducing parent-child conflicts and increasing child compliance and knowledge and decision making skills (3). When parents have correct knowledge but children's knowledge is incorrect, this mismatch in knowledge could cause conflicts. Likewise, parents' knowledge may not be always correct. Without correction, parents' false knowledge could be passed on to their children. It is therefore important to rectify such incorrect knowledge in early years to maximize positive outcomes in children. The Safe City game involves both fun elements and expert-verified, correct safety knowledge which could help children to learn anytime and anywhere. The learning process could*

	also involve parents, such as involving both parents and children to play the game together.<br><br>*References:*<br>1. *Zhang, D., Zhao, J. L., Zhou, L., & Nunamaker Jr, J. F. (2004). Can e-learning replace classroom learning?. Communications of the ACM, 47(5), 75-79.*<br>2. *Furió, D., Juan, M. C., Seguí, I., & Vivó, R. (2015). Mobile learning vs. traditional classroom lessons: a comparative study. Journal of Computer Assisted Learning, 31(3), 189-201.*<br>3. *Miller VA. Parent-Child Collaborative Decision Making for the Management of Chronic Illness: A Qualitative Analysis. Families, systems & health : the journal of collaborative family healthcare. 2009;27(3):249-66.*

**R3(1b):**	**The team highlighted the prevalence of childhood injuries in their recent HCPF-funded project (Ref no.: 26120254) but the paper has not been published yet.**
A:	*Thanks for your comments. We would like to clarify that two papers have been published using the data collected in the HCPF-funded project (Ref no.: 26120254) to examine the prevalence of fall-related injuries among children and adolescent in Hong Kong, and to study the association with stock market fluctuation and rate of self-harm among adolescent in Hong Kong. Both studies have been published in peer-reviewed journals:*<br><br>*Lee, J. C. Y., Tung, K. T. S., Li, T. M., Ho, F. K. W., Ip, P., Wong, W. H. S., & Chow, C. B. (2017). Fall-related attendance and associated hospitalisation of children and adolescents in Hong Kong: a 12-year retrospective study. BMJ open, 7(2), e013724.*<br><br>*Wong, W. H. S., Lee, J. C. Y., Ho, F. K. W., Li, T. M. H., Ip, P., & Chow, C. B. (2017). Stock market fluctuations and self-harm among children and adolescents in Hong Kong. International journal of environmental research and public health, 14(6), 623.*

**R3(1c):**	**Although the investigators adopt the experiential gaming model proposed by Kiili (2005) into their own game model in Annex 1, but there is no description on the figure in the proposal and how it is distinct from the old model.**
A:	*Thank you for your comment.*<br><br>*The experiential gaming model proposed by Kiili (2005) emphasizes the importance of positive user experiences and makes use of immediate feedback, clear goals and developmentally appropriate challenges to maximize the impact of educational games (1). The central tenet of this model is to construct a knowledge base for a specific subject/topic through practice in the game world. By adopting this model, the Safe City game also uses positive user experiences and immediate feedback to change the player's knowledge as illustrated in*

	Annex 1. The difference is that Kiili's model is a general gaming model but our model is specific to safety game. Briefly, the player's journey would begin with identifying the hazard situations based on prior knowledge and cues provided by the game (challenges). Then, the player would pick a solution to removing the hazard (active experimentation). The game system would then tell the player whether his/her solution choice is correct or not (reflective observation). Based on the game feedback, the player would be able to update or confirm the existing base of safety knowledge (schemata construction) which could be used to guide future relevant decision making or behavior. These details have been added in the revised proposal (clean version, P.2).<br><br>*Reference:*<br>*1. Kiili K. Digital game-based learning: Towards an experiential gaming model. The Internet and Higher Education. 2005;8(1):13-24.*

**R3(2):**	**The aims of the proposal are appropriate.**<br><br>**The game design as written in Annex 2, however, is only a game flow similar to those written down by a software game designer. In the background section, the literature about similar game design is lacking. The investigators should write the rationale behind the game from a more philosophical way:**<br><br>**1. Are they using the concept of "Response-cost" by giving them some tokens at the beginning and deduct whenever the child breaks the rule? What is its advantage?**<br>**2. Are they using "Positive reinforcement" whenever the child obeys the safety rule? What is its advantage?**<br>**3. What is the "immerse" level of virtual reality of the game? What is its advantage?**<br>**4. If the designers want to emphasis on following the "stop, look, listen, think" rule, this should be highlighted in the proposal as the main element as it is a cognitive-behavioral therapy (CBT) approach and help the child to make correct decision explicitly through implicitly learning. Apart from this, I also suggest adding "go" and "check" after "think".**<br>**5. Any cueing level? In order to remind the child to achieve the goal in a positive way?**<br>**6. What is the scoring of the game? Any rules for scoring, like correct and incorrect responses, perseveration responses, cues level, reaction time, total time of fame, total points of game, etc.**<br>**7. Any other augmented feedbacks and interactive components? Visual and auditory inputs? Other features like motor which involve actual moving the checkers, etc.**<br><br>**This reflects that the research team does not include experts in task-specific training game design**
A:	*Thank you for the questions. Below please find our response to each question:*

	1. Yes, we would give each player the same amount of coins at the start of the game but when they break the safety rule or give incorrect safety advice, some coins would be deducted from their account which is the same as the concept of "Response-cost". The advantage of using this strategy is to reduce the child's likelihood of future unsafe knowledge/behavior. This advantage information has been added in the revised proposal (clean version, P. 16).<br><br>*2. Yes, we would give coins to the player as rewards when they obey the safety rule or give correct safety advice which is the same as the concept of "Positive reinforcement". The advantage of using this strategy is to increase the child's likelihood of future safety knowledge/behavior. This advantage information has been added in the revised proposal (clean version, P.16).*<br><br>*3. First, we would like to clarify that our proposed game genre is role-playing game, not virtual reality game. Second, as stated in the original proposal, players in our proposed game would create an avatar who will be humans, with various choices for gender, hairstyle, facial features and clothing, to assume the role of powerful "safety inspectors". Previous studies have demonstrated that by creating their own avatar, players will potentially perceive themselves in a first-person game, increasing their immersion into the game world (1). Immersive games are games that can totally capture a player's full attention, as if the player becomes part of the game environment (2) The advantage of being immersive in a game is that immersion is considered a component of intrinsic motivation which means that when a player is immersed, the player would have more positive learning experience and thus greater desire to stay engaged in learning (3). This advantage information has been added in the revised proposal (clean version, P.15).*<br><br>*4. We would like to clarify that the "stop, look, listen, think" rule stated in our proposal is a standard street crossing rule (4). We used it as an example to illustrate how the player could gain or lose point in Safe City. It does not involve any CBT component. We have clarified this in the revised proposal (clean version, P.16).*<br><br>*5. There are some cues in the game. A city map will be given to the player for navigation in the city and planning route to each checkpoint (homes and athletic areas). Once the player enters the checkpoint, the game system would automatically inform the player of the number of unsafe items/behaviors in that particular place as a hint for the player to search. This reminder component has been added in the revised proposal (clean version, P.15).*<br><br>*6. As stated in original proposal, points are automatically added to the player's account for every safe behavior/advice but deducted from their account for every unsafe behavior/advice. In addition, the player will need strategic planning to maximize their points, as places where injuries have occurred because of the inspector's wrong advice will be closed for a short period of time, during which the inspector would have no access to these places to regain the lost money. Further scoring information can be found in Annex 2.*<br><br>*7. First, we would like to clarify that our proposed game genre is role-playing*

	game, not virtual reality game. Feedback would be given to the player in the form of adding or deducting game points which indicates whether the player made a correct or incorrect choice/advice. In addition to the player-item interactions, we would also add in elements to foster interactions between players. For example, players can make friends with other players and exchange items and ideas with each other within the game environment, such that players can work together to solve the problems in the cyber city. This additional social game feature has been added in the revised proposal (clean version, P.16). As for the visual and auditory inputs and other motor features, their quantity and quality would largely depend on the funding amount and hence we would discuss later with the professional game development team after the project is officially launched.<br><br>*References:*<br>*1. You, S., Kim, E., & Lee, D. (2017). Virtually real: exploring avatar identification in game addiction among massively multiplayer online role-playing games (MMORPG) players. Games and Culture, 12(1), 56-71.*<br>*2. Baranowski, T., Buday, R., Thompson, D. I., & Baranowski, J. (2008). Playing for real: video games and stories for health-related behavior change. American journal of preventive medicine, 34(1), 74-82.*<br>*3. Paras, B. (2005). Game, motivation, and effective learning: An integrated model for educational game design.*<br>*4. Zeedyk, M. S., Wallace, L., & Spry, L. (2002). Stop, look, listen, and think?: What young children really do when crossing the road. Accident Analysis & Prevention, 34(1), 43-50.*

**R3(3):**	**The target group is appropriate.**
A:	*Thanks for your comments*

**R3(4):**	**In such a similar training game design study, outcomes can be divided into 2 types: task-specific and domain-specific outcomes. The team identifies task-specific game outcomes as primary outcomes, and domain knowledge such as safety knowledge and clients' self-perceived reflections such as HRQOL and psychosocial difficulties as secondary outcomes.**<br><br>**The secondary outcomes are appropriate but the investigators fail to make sure of the task-specific outcomes.**<br><br>**The task-specific outcomes should be the data obtained from the game which provides big data from more than one thousand cases and the difference in responses and the interactions in different safety domains such as unsafe knowledge, unsafe behaviours, unsafe environments, unsafe management, etc. Other parameters such as correct and incorrect responses, preservation responses, cues level, reaction time, total time of game, total points of game, etc. have not been mentioned in the evaluation. The outcome can also be either reducing the likelihood and consequence of accidents, or both.**

A:	*Thank you very much for the suggestions. First, we would like to clarify that the proposed online city game is 2D with role-playing and interactive elements to reinforce correct safety knowledge in students based on experiential learning theory, instead of training students to practice safety techniques in a simulated environment. To test whether Safe City can achieve this goal, a user log system will be developed to record all in-app activity. Because all player activities will be time-stamped, the time spent on overall and specific parts of the game can be calculated. Total time of game and total points of game have been included in the original proposal under the "Game usage statistics" section. Further, in response to the reviewer's comments, we have revised the proposal to include more task-specific outcomes, including the number of correct and incorrect responses in each safety domain and the number of attempts needed to reach the correct answer (clean version, P. 9) which could help us and other relevant authorities and agencies to identify area(s) within the safety education curriculum that require improvement.*<br><br>*For other suggested parameters such as cues level and reaction time, although they are good indicators of executive functions (e.g. attentional control and cognitive flexibility), these data are more feasible to collect in virtual reality games. In future, if we could secure extra funding to support further development of this game, we would consider upgrading it to a more advanced game such as virtual reality game that would be more able to do both health education and behavioral training for children.*

**R3(5):**	**The research team consists of experts in various fields including injury, child health, etc. but not in game design.**<br><br>**The team does not have previous published papers in this area.**
A:	*We understand the reviewer's concern in our game design expertise, but we would like to highlight our experience in game design for health promotion. First, Professor TC Pong is a senior professor in computer science with extensive research and practice experiences in multimedia computing and e-learning, including gamification of education/health promotion. Second, Dr. Patrick Ip and Dr. CB Chow were the principal investigators of previous HMRF-funded projects on using quiz game competition to reduce smoking and alcohol drinking behaviors. Both studies have been published as indicated below:*<br><br>*Ip P, Lam TH, Chan SSC, Ho FK, ..., Chow CB. Use of Internet Viral Marketing to Promote Smoke-Free Lifestyles among Chinese Adolescents. PLoS ONE. 2014;9(6): e99082.*<br><br>*Ip P, Chan KL, Chow CB, Lam TH, Ho SY, Wong WH, Wong MF. An internet-based intervention to promote alcohol-related attitudinal and behavioral change among adolescents: protocol of a cluster randomized controlled trial. JMIR research protocols. 2016 Apr;5(2).*

**R3(6):**	**The game development fee $780,000 is very expensive. There is no justification about the expenditure as well as item breakdown on this amount in the budget**
A:	*Thank you very much for your comments. Justification and breakdown of the budget item "Game design and development" has been added, as below:*<br>***Game programming: $270,000***<br>*A game programmer will be hired for 12 months to develop codebase for the proposed game.*<br>***Gaming component designing: $280,000***<br>*A game designer will be hired for 12 months to design the game content including creating goals, rules, challenges, game point allocation, level system that could produce a desirable gaming environment for the users to interact and engage in the game.*<br>***Graphics or Art design: $200,000***<br>*A game artist will be hired for 10 months to create 2D art for the visual elements of a video game, such as characters, vehicles, props, scenery, background, objects, colors, textures, and clothing.*<br>***Game sound design: $30,000***<br>*A sound designer will be hired at the final stage of the game design to generate and adjust audio elements for the game.*

**R3(7):**	**The field of childhood injury is an important area but does not receive lots of attention. Promoting safety to reduce accidents in children population is an important and new branch on health promotion.**<br><br>**The idea of game in health promotion in childhood safety is innovative and child-friendly as children like to play smartphone nowadays.**<br><br>**The use of RCT to carry out the experimental intervention (game) by comparing with control group using paper and electronic educational materials is appropriate. However, the proposal does not include a thorough literature on training games, the methodology fails to describe the philosophical design of the game and the domains of childhood safety, and the team is not experienced in designing training games.**
A:	*Thank you for your comments. We have substantially revised the Background and Methodology sections to provide more details to support our proposal. We would also like to clarify that our proposed game is 2D with role-playing game techniques, mini-games and other game elements that foster interactions between players. We are not designing a behavioral virtual reality training game, as such game requires much more time and resources to produce but we would consider this possibility in future development phase. And as clarified earlier, our team has experiences in developing game-based health intervention.*

**R3(8a):**	**Strengths:**<br>**1. Childhood safety is a good area of study in health promotion**<br>**2. The team has a good track record in childhood injury studies**<br>**3. Using game is an innovative idea.**

A:	*Thank you very much for your comments.*

**R3(8b):**	**Weaknesses:**<br>**1. The proposal does not include a thorough literature on training games.**<br>**2. The methodology fails to describe the philosophical design of the game and the domains of childhood safety.**<br>**3. The team is not experienced in designing games.**<br>**4. The task-specific outcome measure is poorly described and utilized which reflected that the team does not have experiences in using training games.**
A:	*Thank you for your comments. We have substantially revised the Background and Methodology sections to provide more details to support our proposal. We would also like to clarify that our proposed game is 2D with role-playing game techniques, mini-games and other game elements that foster interactions between players. We are not designing a behavioral virtual reality training game, as such game requires much more time and resources to produce but we would consider this possibility in future development phase. And as clarified earlier, our team has experiences in developing game-based health intervention. Furthermore, we have revised the proposal to include more task-specific outcomes that are relevant to role-playing games, including the number of correct and incorrect responses in each safety domain and the number of attempts needed to reach the correct answer.*
